# A New Hypnotic

**Published:** 1888-02

**Authors:** 


A New Hypnotic.—Amylen-hydrat (*Dimethylathylcarbinol*, $C_5 H_{12}O$) has been investigated by Prof. J. v. Mering, in Strassburg (*Centralblatt für die gesammte Therapie*, October, 1887), who has found it to be a hypnotic of some value. 15.5 grains dissolved in an ounce of water. It has a somewhat pungent and camphoraceous taste. In physiological action, it resembles paraldehyde, and, in power of effect, ranks between this agent and chloral. Numerically stated, he places them in the following order: Chloral hydrate 1, amylen-hydrate 2, and paraldehyde 3.

Amylen-hydrate can be given by the stomach or by the rectum. Dr. v. Mering advises the following formula for rectal-injection: ℞ Amylen-hydrat. 5; mucil. acaciæ 20; aquæ destil. 50.

For cases of insomnia, due to, or accompanied by pain, he combines morphine with it.

